# Pathology and Advanced Imaging—Characterization of a Congenital Cardiac Defect and Complex Hemodynamics in a Pig: A Case Report

**DOI:** 10.3389/fvets.2021.790019

**Published:** 2021-12-06

**Authors:** Alexandra J. Malbon, Miriam Weisskopf, Lukas Glaus, Sebastian Neuber, Maximilian Y. Emmert, Christian T. Stoeck, Nikola Cesarovic

**Affiliations:** ^1^Institute of Veterinary Pathology, Vetsuisse Faculty, University of Zurich, Zurich, Switzerland; ^2^Center for Surgical Research, University of Zurich, University Hospital of Zurich, Zurich, Switzerland; ^3^Translational Cardiovascular Technologies, Department of Health Sciences and Technology, Swiss Federal Institute of Technology, ETH Zurich, Zurich, Switzerland; ^4^Cardiosurgical Research Group, Department of Cardiothoracic and Vascular Surgery, German Heart Center Berlin, Berlin, Germany; ^5^Translational Cardiovascular Regenerative Technologies Group, Berlin Institute of Health at Charité – Universitätsmedizin Berlin, BIH Center for Regenerative Therapies, Berlin, Germany; ^6^Institute for Regenerative Medicine, University of Zurich, Zurich, Switzerland; ^7^Institute for Biomedical Engineering, Department of Information Technology and Electrical Engineering, Swiss Federal Institute of Technology, ETH Zurich, Zurich, Switzerland

**Keywords:** large animal models, cardiovascular imaging, congenital heart defects, cardiovascular pathology, atrial septal defect, blood flow

## Abstract

Domestic pigs are widely used in cardiovascular research as the porcine circulatory system bears a remarkable resemblance to that of humans. In order to reduce variability, only clinically healthy animals enter the study as their health status is assessed in entry examination. Like humans, pigs can also suffer from congenital heart disease, such as an atrial septal defect (ASD), which often remains undetected. Due to the malformation of the endocardial cushion during organ development, mitral valve defects (e.g., mitral clefts) are sometimes associated with ASDs, further contributing to hemodynamic instability. In this work, we report an incidental finding of a hemodynamically highly relevant ASD in the presence of incompetent mitral and tricuspid valves, in an asymptomatic, otherwise healthy juvenile pig. In-depth characterization of the cardiac blood flow by four-dimensional (4D) flow magnetic resonance imaging (MRI) revealed a prominent diastolic left-to-right and discrete systolic right-to-left shunt, resulting in a pulmonary-to-systemic flow ratio of 1.8. Severe mitral (15 mL/stroke) and tricuspid (22 mL/stroke) regurgitation further reduced cardiac output. Pathological examination confirmed the presence of an ostium primum ASD and found a serous cyst of lymphatic origin that was filled with clear fluid partially occluding the ASD. A large mitral cleft was identified as the most likely cause of severe regurgitation, and histology showed mild to moderate endocardiosis in the coaptation area of both atrio-ventricular valves. In summary, although not common, congenital heart defects could play a role as a cause of experimental variability or even intra-experimental mortality when working with apparently heathy, juvenile pigs.

## Introduction

Due in part to their good availability, acceptable cost, and relatively easy handling, domestic (farm) pigs are often used in research (1). The similarities between the cardiovascular anatomy and physiology of pigs and humans make them a valuable model-species in cardiovascular science projects. In order to reduce inter-animal variability, but also to exclude the effects of comorbidities on the experimental read-out, only clinically healthy animals are allowed to participate in a scientific study. However, thorough, pre-entry clinical examinations are usually focused on infectious diseases or apparent malformations. It is thereby extraordinarily challenging to ascertain the state of cardiac health in large laboratory animals such as pigs, especially during entry examination. In this context, congenital heart defects may go undetected, particularly in juvenile animals, which often show no obvious signs of distress or apparent stunted growth. Atrial septal defects (ASDs) are among the three most common types of congenital heart disease in pigs (2). The same study found a prevalence for ASD of 1.6 per 100 live births in pigs, whereas it has an estimated prevalence of 1.6 per 1,000 live births in humans (3). ASDs include several distinct types of atrial communication and allow shunting of blood between the systemic and the pulmonary circulations (4). The flow direction of the shunted blood is often determined by ventricular compliance. As the right ventricle (RV) is more compliant than the left, large ASDs are often associated with prominent left-to-right (L-R) shunting and result in right ventricular volume overload and reduced left ventricular output (4). If left untreated, patients with large ASD will gradually develop right ventricular dilatation and eventually right-sided heart failure. Mitral and/or tricuspid regurgitation has been observed in some cases, as atrioventricular valves are often malformed in the presence of ostium primum ASD (5). However, in large animals used in cardiovascular research, the presence of complex congenital cardiac malformations is rarely described.

In order to diagnose and delineate congenital heart defects in large laboratory animals as well as in the clinic, multiple imaging modalities can be used, with echocardiography as a primary screening tool (6). Furthermore, magnetic resonance imaging (MRI) is increasingly gaining acceptance and importance in ASD diagnosis and follow-up care (7). Particularly for blood flow imaging, phase contrast MRI is becoming an increasingly important method in cardiovascular medicine and research (8). However, none of these imaging techniques are routinely used in clinical pre-assessment of large laboratory animals, such as pigs. Furthermore, it is relatively rare that intravital cardiac imaging results can be correlated with pathological findings. Here, we present a case report in which detailed imaging analysis was combined with a pathological examination.

## Case Description

All pigs entering the research facility are provided by the same supplier and screened under the national surveillance program for classical and African swine fever, foot and mouth disease, Aujeszky's disease, porcine reproductive and respiratory syndrome (PRRS) and swine vesicular disease. While still at the supplier, piglets are vaccinated against *Glaesserella parasuis* and porcine circovirus at the age of 3 and 6 weeks, whereas sows are vaccinated against Parvovirus and *Erysipelothrix rhusiopathiae*. Additionally, sows are vaccinated against *E. coli* 5 and 3 weeks prior to giving birth. Upon arrival at our facility, all pigs are clinically assessed by observation (general behavior, posture and gait, visible injuries, color of the skin, breathing pattern, nasal and ocular discharge, appetite, defecation and urination) and only if further indicated by physical examination as it often induces additional stress to newly arrived animals. Following an unremarkable entry examination, a 30 kg female pig (Swiss White large “Edelschwein”) ~10–12 weeks old, was included in an acute study as part of a scientific project investigating the use of hyperpolarized 13C urea for magnetic resonance perfusion imaging under the license ZH152/2013. After 10 days of habituation, the animal was anesthetized for the imaging procedure as part of the experimental protocol. General anesthesia was induced by intramuscular application of ketamine (15 mg/kg), azaperone (2 mg/kg) and atropine (0.05 mg/kg), deepened with intravenous propofol (1 mg/kg) for orotracheal intubation and maintained with isoflurane (2–3%) in 100% oxygen under volume controlled, positive pressure ventilation (tidal volume 8–10 mL/kg; respiration frequency 20 bpm; PEEP 5–6 mmHg; Pmax 25 mmHg). The animal was subjected to functional and phase contrast MRI (2 × 2 × 2 mm spatial resolution, 42 ms temporal resolution) on a clinical grade 1.5 T Philips Ingenia scanner (Philips Healthcare, Best, The Netherlands). Upon completion of the imaging procedure, the animal was euthanized with an i.v. overdose of pentobarbital (150 mg/kg; Esconarkon^®^, Streuli Pharma AG, Uznach, Switzerland) while still in deep anesthesia; according to the study protocol. The heart was harvested and preserved in 10% neutral buffered formalin for pathological analysis. Following macroscopic examination, tissue blocks of the left and right atria, left and right ventricular free walls, and the septum were prepared and histological sections of the affected regions were made. Tissue blocks were routinely processed and embedded in paraffin wax prior to haematoxylin and eosin staining of 2 μm sections. Consecutive sections were additionally stained with reticulin, elastic van Gieson and trichrome stains or mounted onto positively charged glass slides and stained for Factor VIII-related antigen (FVIIIra) and CD31 using immunohistochemistry.

## Diagnostic Assessment

### Imaging Results

**Figure 1:**
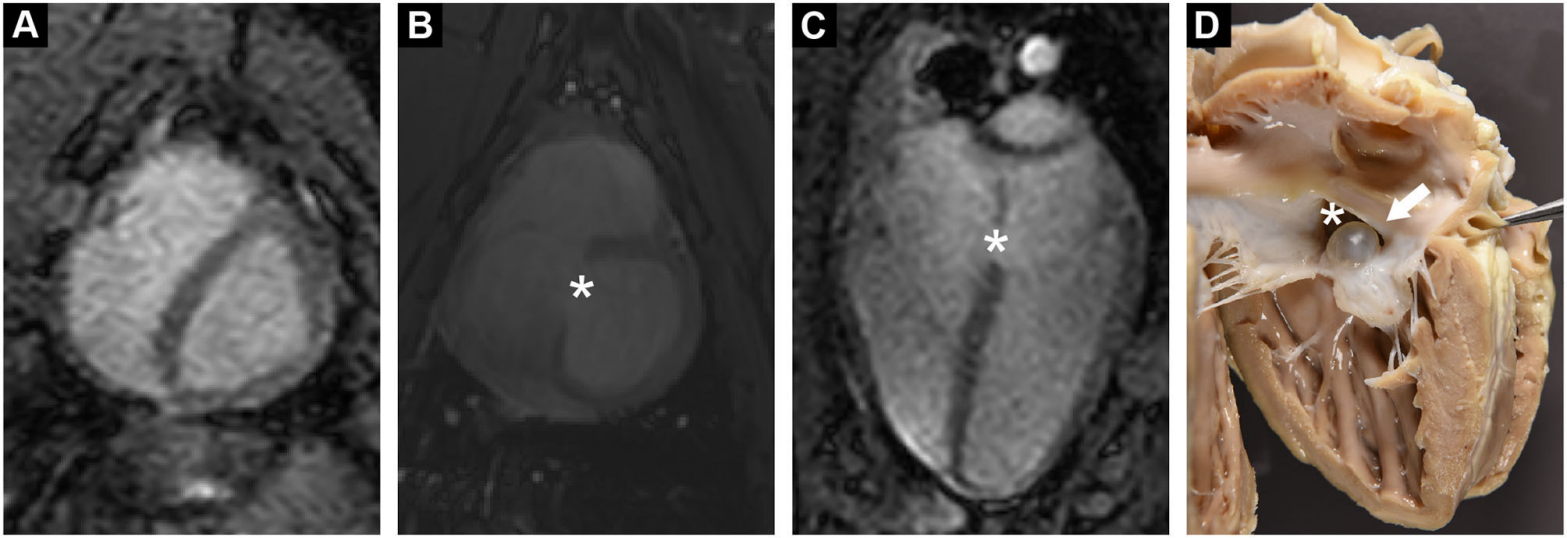
**(A)** Functional cardiac MRI in short axis revealed a diastolic D-shaped left ventricle, indicating increased right ventricular pressure. **(B,C)** Cardiac MRI in short and long axis demonstrated a tissue defect in the inter-atrial septum (*). **(D)** Post mortem examination confirmed a large ASD (*). ASD was partially occluded by a serous cyst (arrow).

**Figure 2:**
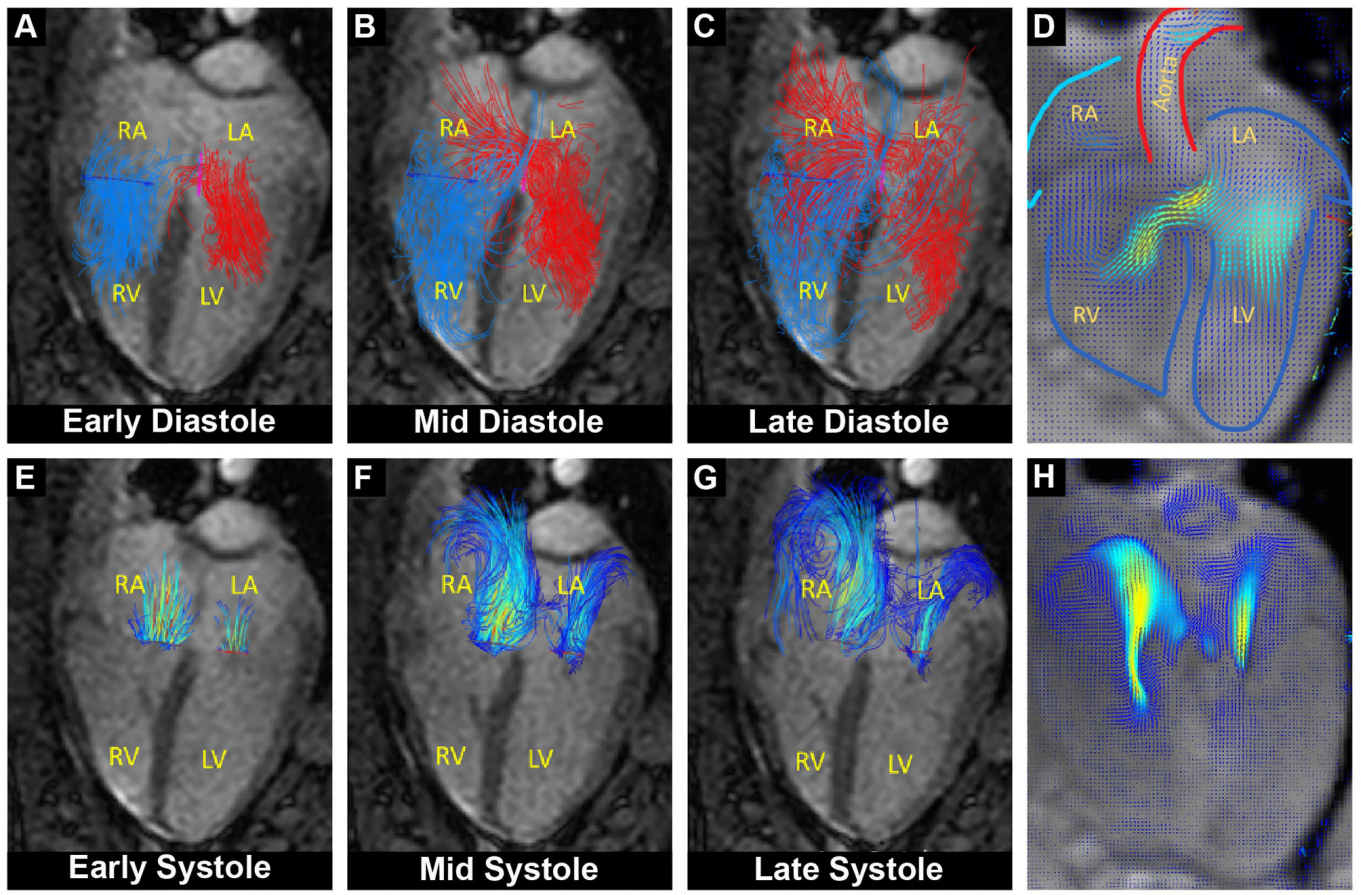
Overlay of cardiac functional MRI and pathline visualization of blood flow during the cardiac cycle. RA, right atrium; LA, left atrium; RV, right ventricle; LV, left ventricle. **(A–C)** Pathline visualization of blood flow originating from the left (red) and from the right (blue) atrium, showing left-to-right flow across the atrial septum during diastole (L-R shunt). **(D)** Velocity field visualization focused on the diastolic shunt across the atrial septum. **(E–G)** Pathline visualization of regurgitant blood flow through the closed mitral and tricuspid valve during systole. **(H)** Velocity field visualization focused on the systolic mitral and tricuspid regurgitant flow.

Functional MRI showed a clear defect in the atrial septum and a moderately enlarged right ventricle (Figures 1A–C). Analysis of the phase contrast MRI data performed on specialized GTFlow software (Gyrotools) revealed a diastolic L-R and a systolic right-to-left (R-L) shunt through the atrial septal defect, as well as mitral and tricuspid valve insufficiency and disturbed diastolic intra cardiac blood flow patterns. The systolic R-L shunt was discrete and had a total volume of 4.6 mL per stroke with a maximum jet velocity of 25 cm/s. Peak diastolic L-R shunt velocity through the ASD was 48 cm/s and the total volume of shunted blood was 14.1 mL per stroke. Peak velocities of mitral and tricuspid regurgitant jets were 36.4 and 46.9 cm/s, respectively, indicating a severe dysfunction of the atrioventricular (AV) valves (Figure 2). Surprisingly, mitral back-flow (regurgitation) was observed both during systole and diastole, with a total regurgitant volume of 15 mL per stroke (990 mL/min). Moreover, end-diastolic D-shape of the left ventricle was observed, indicating right ventricular volume overload (Figure 1A). Using blood particle tracking analysis, we were able to estimate that 26% of the blood volume contained in the diastolic mitral regurgitation (MR) jet directly crossed the ASD orifice and reached the RV, while only 18% of the systolic MR jet volume crossed the ASD in the subsequent diastole. Tricuspid regurgitation was limited to systole only, but was more severe with a total regurgitant volume of 22 mL per stroke (1.4 L/min).

Furthermore, MRI examination revealed an elevated ratio of pulmonary-to-systemic flow (Qp/Qs = 1.8), supporting the presence of a hemodynamically relevant ASD (area of 182 mm^2^) with a diastolic L-R shunt (930 mL/min).

### Pathology Results

**Figure 3:**
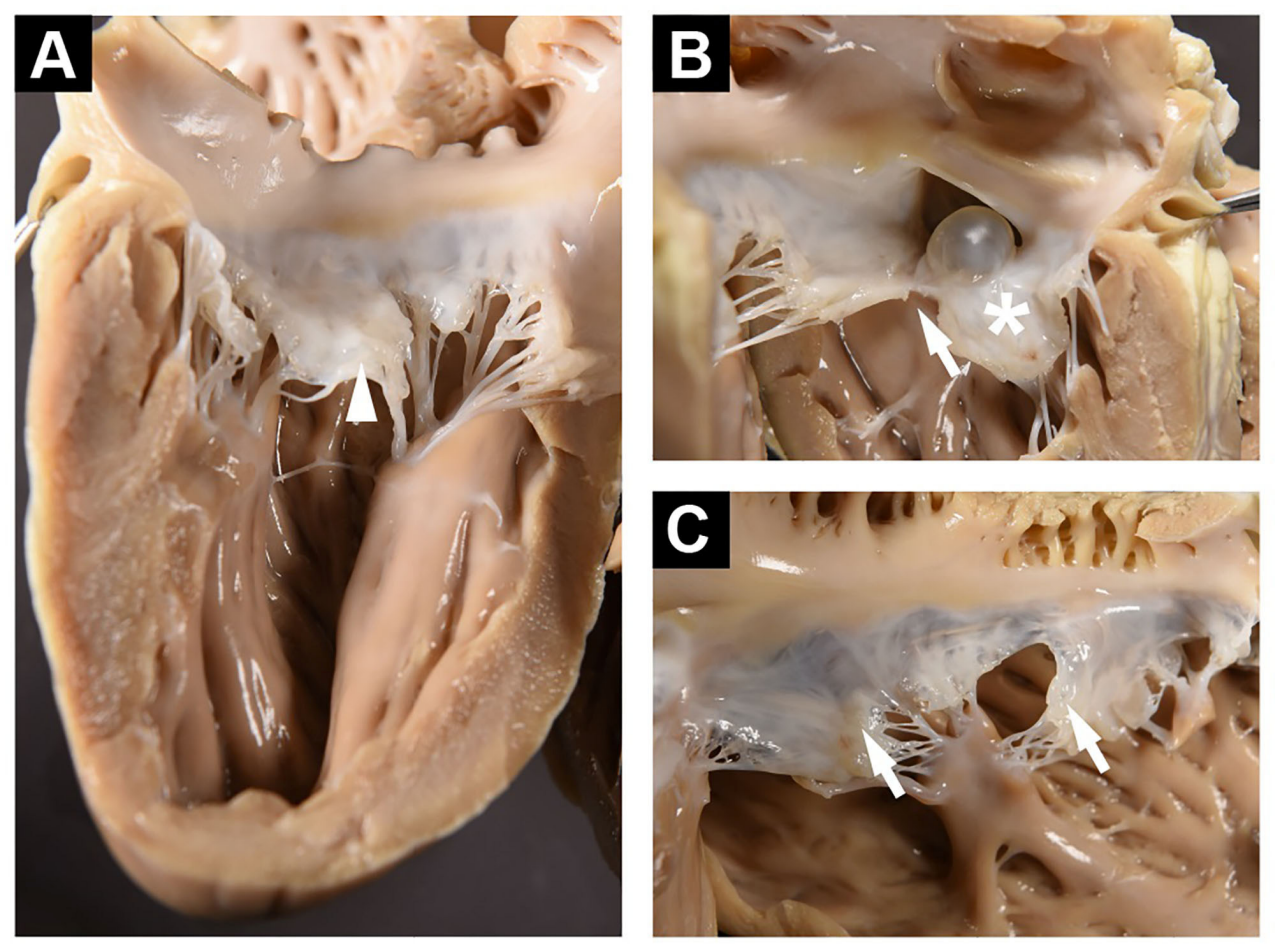
**(A)** Left ventricular free wall and posterior mitral valve showing enlarged middle scallop (arrowhead). **(B)** Septum as viewed from the left ventricle showing ASD and cyst. The anterior mitral leaflet is exhibiting diffuse thickening (*), and A1/A2 clefting (arrow). **(C)** Right ventricular free wall and tricuspid valve with small nodular thickenings at the free margin (arrows).

**Figure 4:**
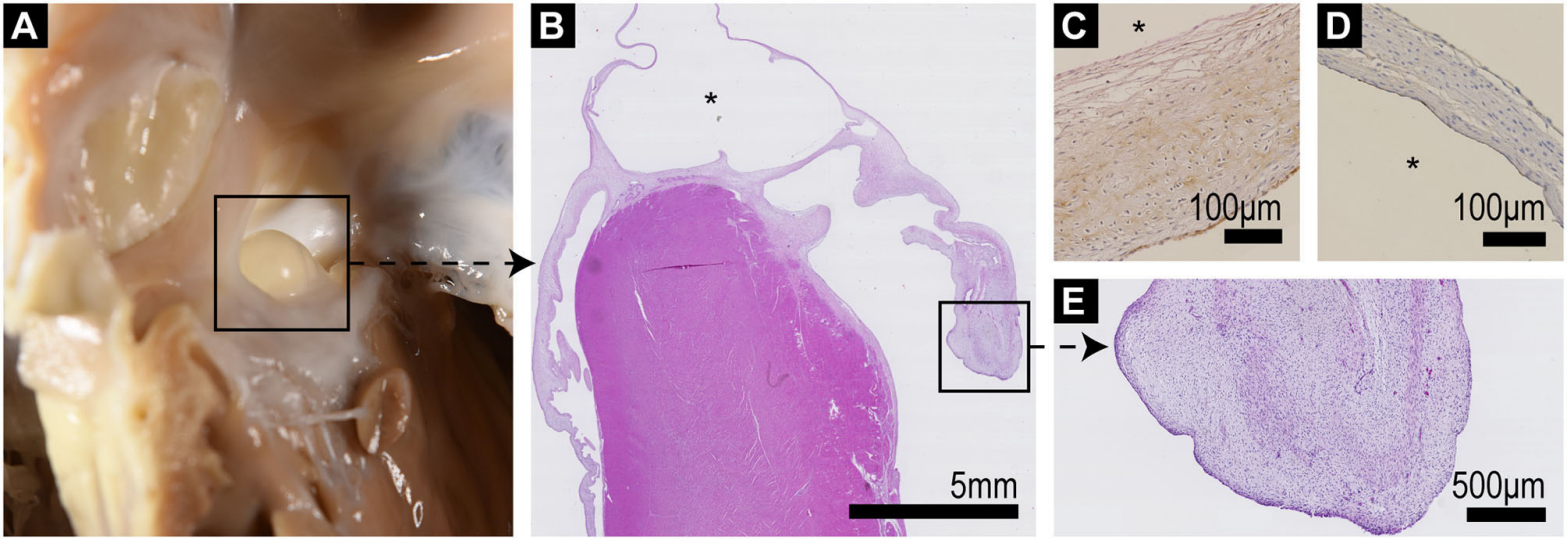
**(A)** Septum with ASD and protruding serous cyst as viewed from the right ventricle. **(B)** Low power (10x) histological overview of a longitudinal section through the interventricular septum including the cyst (*) which bridges the septum and atrioventricular valves; the left AV valve is to the right of the image, exhibiting marked thickening and distortion of the valve leaflet particularly at its tip. **(C)** FXIIIra/vWF staining shows positive staining of the outer aspect of the cyst wall, which corresponds to the endocardial valve surface, with negative staining of the inner (*) lining. **(D)** CD31 (pan-endothelial marker) staining shows positive staining of both aspects of the cyst wall. **(E)** Higher power view of the thickened valve tip of the mitral valve showing loss of layering of the leaflet and expansion of the spongiosa by whorls of stellate cells within a loose pale staining proteoglycan matrix (endocardiosis).

The autopsy of the heart revealed a defect in the atrial septum in which the atrioventricular orifices remained separate, but with a continuous AV valve bridging the septum, thus creating an ostium primum ASD (Figures 1D, 3B). The membranous oval fossa was prominently delineated, but the foramen ovale was not patent. The mitral valve showed a large cleft in the anterior leaflet segment A1/A2, with A1 partially connected to the ventricular wall (Figure 3). Both mitral leaflets, especially the anterior, were diffusely thickened. The tricuspid valve also had several mild nodular thickenings at its free margin (Figure 3). Histologically, these changes corresponded to a loss of valvular layer definition, with a disruption of the fibrosa and an expansion of the spongiosa. The spongiosa layer showed reduced staining uptake due to the deposition of oedematous ground substance and extracellular matrix components surrounding loosely arranged whorls of stellate cells. These findings indicate myxomatous valvular degeneration (endocardiosis) (Figure 4C). A serous cyst with a diameter of 1 cm protruded into and partially occluded the septal defect, extending from the septum at the exposed junction of the left and right AV valves (Figure 4A). Histologically, the cyst was lined by a flat endothelial cell layer. Immunohistochemical staining of the cyst revealed a positive reaction of the cyst wall with CD31 and a negative reaction with FVIIIra, which corresponds to lymphatic origin and is compatible with the clear fluid contents of the cyst (Figure 4).

## Discussion

Animal pre-selection and health screening are an essential part of every pre-clinical trial. However, health examination of farm pigs entering scientific studies are challenging to perform and are often focused on general health and preventing infectious diseases from entering the facility. As a result, some cardiac conditions might go undetected. This is especially true for congenital heart defects in juvenile farm pigs, as in this age group the heart condition can still be effectively compensated with animals displaying no obvious symptoms (9). In the present study, this was the case for a juvenile female pig with an incidental finding of ASD with highly relevant L-R shunt, severe mitral and tricuspid regurgitation, and an increased pulmonary-aortic flow ratio.

The type of ASD described in this study is part of the spectrum of atrioventricular septal defects (AVSDs) and is also known as incomplete AVSD or endocardial cushion defect.

AVSDs account for ~3% of congenital cardiac defects in humans and are also highly associated with both Noonan and Down syndrome (10). The most common type of ASD is the ostium secundum defect, which accounts for ~80% of ASDs seen in the clinic. This defect represents a tissue deficiency of the fossa ovalis. Ostium primum ASDs, as in our case, account for only about 10% of septal defects and develop owing to a deficiency in tissue near the atrioventricular valves (11). Ostium primum ASDs are often associated with clefting of the mitral valve, potentially inducing severe regurgitation. Although present at birth, ASD are often only diagnosed in adulthood as patients are frequently asymptomatic in their early life (11). Despite late development of symptoms, large ASDs induce dramatic circulatory perturbations and treatment is usually indicated (12). Conversely, it has been shown that AVSD and ASDs represent the most frequent congenital cardiac anomalies in farm pigs as well as in cloned and transgenic piglets (2, 13, 14). Moreover, heritable ventricular septal defect syndrome has been reported in Yucatan miniature swine breed for scientific purposes (15). However, little is known about the development and progression of AVSD and ASDs in farm pigs, as they are often presented as slaughter-house findings (2).

Diastolic L-R shunting as described in this case could be attributed to hemodynamic effects caused by differences in ventricular stiffness and compliance, whereas the small systolic R-L shunt could be due to pulmonary hypertension and severe tricuspid regurgitation. Diastolic MR is a rare event and often indicates diastolic left ventricular dysfunction or AV block (16). Furthermore, our case showed that the association of ostium primum ASD and mitral valve clefts seen in humans can also be applied to farm pigs. This valvular defect was probably the cause of severe mitral regurgitation that further decreased the forward cardiac output. Furthermore, myxomatous degeneration of the atrio-ventricular valves shown in the presented case, is often seen in patients with incompetent valves (17). Often described in dogs as a major contributor to mitral regurgitation, myxomatous valvular degeneration is though quite unusual in juvenile animals as in our case (18).

Cysts associated with ASD are rare but have been reported in patients previously. However, they were often either tumorous (myxoma) in origin or blood cysts usually filled with necrotic tissue and thrombus material (19, 20). Valvular cysts are relatively common in domestic farm species, particularly cattle, and are usually found as an incidental finding at slaughter (21). They may be of blood or lymphatic vascular origin. Approximately 2% of pigs may also be affected, although it rarely appears in animals under 6 months of age (22). Such valvular cysts are often filled with clear, aqueous fluid and lined with cells indistinguishable from the valvular endothelium (22). The age of the animal in our case was unknown as only the weight was available, but can be estimated at 2–3 months.

Hence, congenital septal and related valvular defects in humans as well as in pet and farm animals are quite similar in anatomical appearance and corresponding hemodynamic effects. Although important, diagnosis of such cardiac disease could be quite challenging in large laboratory animals. Specialized imaging techniques such as functional and phase contrast MRI, besides echocardiography, can be applied. However, such MRI procedures require general anesthesia with multiple breath holds and are lengthy. Nevertheless, modern acquisition sequences and hardware are available that can significantly shorten the scan time and hence increase safety and ‘comfort' of large animals used for scientific purposes (23, 24).

## Conclusion

Domestic (i.e., farm) pigs are widely used in studies of human heart disease and therapy, mainly due to the similarity in heart development, physiology, and anatomy. These similarities also extend to congenital heart defects, such as ASD. Although having a strong impact on cardiac function, such defects are often not apparent upon clinical examination prior to the study and their diagnosis often require specialized imaging procedures like echocardiography or magnetic resonance. Easier to perform, jet less specific diagnostic methods, like cardio-pulmonary auscultation, might be successfully used with well-adapted and/or trained (mini-) pigs, however they are quite challenging in a setting of entry examination of farm pigs not used to human handling. Although echocardiographic equipment is widely available in the laboratory animal setting, examinations still require pigs to be sedated. Transthoracic as well as transoesophageal echo-imaging windows are quite restrictive in porcine and a comprehensive echocardiographic examination requires experience and adapted imaging protocols (25). On the other hand, magnetic resonance imaging of cardiac morphology, function and blood flow as well as myocardial perfusion and microstructural changes (such as fibrosis) can readily be obtained in farm pigs used for scientific purposes (26). By using standardized human-grade MRI protocols, ASD was successfully uncovered in this case. However, such protocols require specialized equipment, additional procedural handling and general anesthesia, hence are often not applicable in a routine laboratory setting.

Hence, congenital heart defects should be considered as a possible cause of inter-animal variability or even mortality when working with healthy-appearing juvenile domestic pigs in scientific projects. However, the detection of such defects is challenging and requires specialized imaging procedures *in vivo* or detailed pathological post-mortem examination.

## Data Availability Statement

The raw data supporting the conclusions of this article will be made available by the authors, without undue reservation.

## Ethics Statement

The animal study was reviewed and approved by Cantonal Veterinary Office Zurich.

## Author Contributions

NC, MW, and CS: conceptualization, imaging procedure, animal handling, data reconstruction, data analysis, and preparation and review of manuscript. AM and LG: pathology data acquisition, data analysis, and preparation of the manuscript. SN and ME: data analysis, data interpretation, and review of manuscript. All authors contributed to the article and approved the submitted version.

## Funding

This work was partially funded by grants from the Swiss National Science Foundation (PZ00P2_174144 and CR23I3_166485) and Olga Mayenfish foundation.

## Conflict of Interest

The authors declare that the research was conducted in the absence of any commercial or financial relationships that could be construed as a potential conflict of interest.

## Publisher's Note

All claims expressed in this article are solely those of the authors and do not necessarily represent those of their affiliated organizations, or those of the publisher, the editors and the reviewers. Any product that may be evaluated in this article, or claim that may be made by its manufacturer, is not guaranteed or endorsed by the publisher.
